# “If it weren’t for COVID-19…”: Counterfactual arguments influence support for climate change policies *via* cross-domain moral licensing or moral consistency effects

**DOI:** 10.3389/fpsyg.2022.1005813

**Published:** 2022-11-10

**Authors:** Mauro Bertolotti, Luca Guido Valla, Patrizia Catellani

**Affiliations:** Department of Psychology, Catholic University of Milan, Milan, Italy

**Keywords:** COVID-19, climate change, counterfactual communication, moral licensing, moral credits, moral consistency

## Abstract

In two studies, we investigated whether counterfactual messages (i.e., “If… then…”) on the economic costs of past public policies influence support for future climate change policies. In Study 1, we tested whether the effect of upward counterfactual messages depended on their referring (or not) to the COVID-19 pandemic. Results showed lower support for a future climate change policy when the past expenses evoked by the upward counterfactual messages were attributed to COVID-19. In Study 2, we combined upward counterfactuals with downward counterfactuals presenting past economic efforts to deal with the COVID-19 pandemic as a moral credit. Results showed that exposure to downward counterfactuals decreased support for climate change policies among participants with low endorsement of anti-COVID-19 measures, whereas it increased support among participants with high endorsement. Discussion focuses on the conditions under which counterfactual communication may activate cross-dimensional moral licensing or moral consistency effects, influencing support for climate change policies.

## Introduction

Since its emergence in early 2020, the COVID-19 pandemic has understandably monopolized public opinion and collective economic efforts to tackle it. This threat to citizens’ health and safety, the sweeping measures that national governments have adopted to address it, and their economic repercussions, have cast some shadow on other relevant and urgent problems, such as global warming and climate change, and might continue to affect the way the public sees them. A YouGov poll conducted in 11 European countries showed that where the economy was most hardly hit by the pandemic, or where economic conditions were already precarious, such as in Spain, Portugal, Greece, and Italy, a large proportion of citizens (from 58 to 66%) ranked economic recovery from the COVID-19 pandemic as a higher priority than environmental sustainability (YouGov, 2020). Consistently, past research (Ecker et al., 2020) found that framing climate change as a secondary issue during the pandemic emergency reduces citizens’ environmental concern and support for mitigation policies. As the consequences of the COVID-19 continue to affect the economic conditions of many countries, some politicians and actors in the public discourse might argue against the adoption of pro-environmental policies stating that climate change policies could have been adopted, if it were not for COVID-19 and its repercussions on the economy. Some citizens might find these arguments convincing and use past economic turmoil as an excuse not to support future collective commitment to tackle climate change.

In this paper we investigated whether communication on COVID-19 influences support for climate change policies. Specifically, in two studies we analyzed for the first time whether an upward counterfactual (i.e., “If only…”) statement on past public expenses to curb the spread of the pandemic can be used as an excuse for withdrawing support for a future climate change policy, and whether downward counterfactual statements on public health outcomes can strengthen or hinder the persuasiveness of such excuse.

## Theoretical background

### The effects of communication on the economic costs of climate change policies

Past research has shown that citizens’ support for climate change policies is affected by communication on their expected costs (DeGolia et al., 2019) and benefits (Bain et al., 2012; Bertolotti and Catellani, 2014; Stecula and Merkley, 2019). Politicians and interest groups opposing climate change policies sometimes exploit the persuasiveness of economic arguments, framing the adoption of environmental policies as a trade-off between future environmental benefits and future economic costs or losses (Ecker et al., 2020). Economic arguments have been shown to be effective not only with those who are already against the climate change policy (Whitmarsh and Capstick, 2018; Leiserowitz et al., 2020), but also with those who agree with the policy in principle (Bertolotti et al., 2021).

Economic arguments against the adoption of climate change policies are often formulated in *prefactual* terms focusing on the anticipated negative outcomes of these policies (e.g., “If we regulate carbon emissions, we will impose excessive burdens on companies in the energy and manufacturing sectors”). Prefactuals are conditional propositions (if… then…, Byrne and Egan, 2004; Epstude et al., 2016) simulating how present or future actions and decisions can lead to a certain outcome in the future. Past research has shown the persuasive force of a prefactual communication of this type (Bertolotti and Catellani, 2014, 2015; Bertolotti et al., 2021). But economic arguments against climate change policies may be also formulated in *counterfactual* terms, focusing on how past events could have made their adoption more feasible in the present (e.g., “If the economy had been in better conditions, we would have been able to make investments in renewable energy sources”). Counterfactuals (Roese, 1997; Catellani and Covelli, 2013) are conditional propositions simulating how things could have been different if some element of the past had been altered. Individuals can mentally simulate either *better* or *worse* alternatives to reality, thus formulating *upward* or *downward* counterfactuals, respectively (Sanna, 1998; Eisma et al., 2021).

Past research indicates that counterfactuals partially obfuscate the persuasive intent of the speakers and allow them to make statements without fully committing to the hypothetical scenarios they propose (Fiedler and Mata, 2013), thus preventing possible backlash for negative or controversial statements (Catellani and Bertolotti, 2014; Bertolotti and Catellani, 2018). By establishing a link between a policy under discussion and an unmodifiable past event, a counterfactual statement is likely to reduce the need for the speakers to justify their position against the adoption of the policy, blaming said past event for it.

The persuasiveness of an economic argument against the adoption of climate change policies might be therefore enhanced by claiming that the policy could have been adopted “if only…” the economic conditions were different, rather than by directly rejecting the policy itself. No empirical studies have tested this hypothesis, so far. In the present research we explored whether the COVID-19 pandemic and its related exceptional public expenses provide an adequate basis for counterfactual arguments against the adoption of climate change policies.

### Upward counterfactuals focused on COVID-19 as an excuse to not support climate change policies

Counterfactual thinking is often triggered by events that are perceived as a deviation from the routine (Kahneman and Miller, 1986) or social norms (Catellani and Milesi, 2001; Catellani et al., 2004; Catellani and Milesi, 2005; Halpern and Hitchcock, 2015). When individuals detect a deviation from normality, they mentally simulate how things would have gone if a supposedly disruptive element was removed. In doing so, they focus on a specific element or actor in the event (Medvec et al., 1995; Gerstenberg et al., 2012; Alicke et al., 2015), attributing it a prominent causal role (McClure et al., 2007), and a certain degree of responsibility for the final outcome (Zultan et al., 2012; Catellani et al., 2021). Another factor that often triggers counterfactual thinking is the negative valence of events and outcomes, as individuals are motivated to figure out how things could have been better if only things had gone differently in the past. Past research has shown that when people experience negative outcomes, or reflect on them, they tend to spontaneously generate more *upward* counterfactuals, i.e., hypothetical comparisons with how things would have gone *better* otherwise, than *downward* counterfactuals, i.e., hypothetical comparisons with how things would have gone *worse* (Kahneman and Miller, 1986; Mandel, 2003).

Since exceptional and negative events tend to trigger upward counterfactuals, an event such as the COVID-19 pandemic has likely made such counterfactual thoughts highly available to individuals’ minds across a range of situations, including when public policies are discussed. As several countries were in the process of discussing their plans for climate mitigation right when the pandemic struck, the heightened availability of counterfactual thoughts might be exploited to explain decisions taken (or not taken) regarding climate change policies. In this vein, Ecker et al. (2020) presented participants with a fictional newspaper article arguing that given the sudden and extreme negative consequences of the pandemic on the economic situation, climate change policies should take a “back seat” for a while. Such argument implicitly hints at a counterfactual scenario (i.e., “If the pandemic had not occurred, we could have focused on climate change”) and the results of this study showed that exposure to this argument indeed resulted in decreased concern for climate change and lower support for mitigation policies. A temporary disengagement from the climate change issue was therefore apparently excused by the global health emergency.

Past research indicates that upward counterfactual statements can be effectively employed as excuses (Wong, 2010; Catellani and Bertolotti, 2014). For example, they can effectively influence recipients’ evaluations of politicians’ past behavior, resulting in more lenient attributions of responsibility for their inappropriate or insufficient action. Politicians can effectively defend their poor results by saying that things would have gone better, if the conditions had been different or if the opposition had not countered their efforts (Catellani and Covelli, 2013; Catellani and Bertolotti, 2014). Further research showed that individuals use counterfactual thinking to excuse their past failures and maintain a positive self-esteem (McCrea, 2008). Crucially, counterfactual excuse has also the effect of reducing one’s commitment to a task or goal and the intention to achieve better results in the future (Mercier et al., 2017; Smallman and Summerville, 2018). Upward counterfactual arguments focusing on an exceptional event, such as the COVID-19 pandemic, may therefore have a similar effect on citizens’ support for future collective efforts, as well.

### Downward counterfactuals focused on COVID-19 as triggers of moral licensing versus moral consistency

Upward counterfactual excuses of the type described above may be accompanied by downward counterfactuals stating that the consequences of the COVID-19 would have been even *worse* if the public expenses to curb the spread of the virus had not been made. Exposure to downward counterfactuals of this type might moderate the effect of the upward counterfactual excuses, *via* the activation of a moral credit (Nisan, 1991). Focusing on the collective efforts undertaken in the recent past and the negative outcome they prevented (in this case greater spread of the virus, more hospitalizations, and eventually more deaths), downward counterfactuals might provide a moral credit which individuals may be induced to “spend” by retaining their support for climate change policies. This possibility is consistent with the results of past research on *moral licensing* (Monin and Miller, 2001), namely, the tendency to use past (moral) actions to excuse present and future inaction (or immoral action).

According to the theory of moral licensing, moral choices made in the past may license individuals to engage in immoral or unethical behaviors in the future. This is due to the human propensity to reduce uncertainty by anchoring morality judgments (and self-judgments in particular) to past virtuous behaviors (Merritt et al., 2010; Merritt et al., 2012). Using the metaphor of a moral bank account, individuals gain moral credits (Nisan, 1991) by performing good deeds and spend them by committing bad deeds (Hollander, 1958). By acquiring and maintaining a positive moral balance, individuals feel licensed to engage in questionable or immoral acts. To do so, they often engage in a process of selective and strategic recall, remembering past virtuous actions to be indulgent with themselves, and feel more justified when they engage in subsequent immoral behaviors (Effron et al., 2009; Mazar and Zhong, 2010; Jordan et al., 2011; Conway and Peetz, 2012).

Counterfactual thinking can be used as a subtle strategy to acquire moral credits (Effron, 2014), claiming them not from actual moral actions, but from the immoral actions that one could have pursued in the past. In other words, individuals can evoke an “*immoral road not taken”* by mentally simulating an immoral choice they avoided in the past, to acquire a moral credit in the present or the future (Effron et al., 2012, 2013). So far, research has investigated counterfactual thinking as an intra-personal process that may be related to moral licensing (Blanken et al., 2015; Simbrunner and Schlegelmilch, 2017 for meta-analytical reviews). In the present research, for the first time we explored the possibility that exposure to counterfactual communication can also trigger moral licensing. We tested whether reading a downward counterfactual message, describing how the COVID-19 pandemic would have had worse consequences if adequate public expenses had not been undertaken, may influence recipients’ support for public expenses in another domain, that is, the mitigation of climate change.

Past environmental research has already detected *intra-domain* moral licensing (Tiefenbeck et al., 2013; Truelove et al., 2014; Gholamzadehmir et al., 2019), while only few studies have investigated *inter-domain* moral licensing (e.g., Mazar and Zhong, 2010; Miller and Effron, 2010), that is, tested whether a person’s moral behavior in one domain is used to justify less moral behavior in another domain. However, it should be noted that this stream of research has gained momentum in the recent literature, as evidenced by recent works on intra- vs. inter-domain effects (Reimers et al., 2022). So, in the present research for the first time we tested whether decisions in the environmental domain may be influenced by counterfactual reference to past decisions in another domain, namely, the public health domain. Moral licensing, however, is just one possible outcome of evoking past moral behavior. When individuals are strongly identified with a moral cause, recalling past related behavior may make commitment salient, and consequently motivate them to act in a consistent way to uphold their moral identity (Shao et al., 2008). Therefore, in the case of people who are highly committed to a moral cause, recalling a past collective moral behavior may induce a *moral consistency* effect, that is, an increased (rather than decreased) likelihood of engaging in further moral behaviors (Conway and Peetz, 2012; Mullen and Monin, 2016). It should be noted that past literature has highlighted some limitations in the application of the moral licensing theory. For instance, Blanken et al. (2015) highlighted that the effect size and the conditions under which the moral licensing effects take place are still uncertain. Moreover, according to Lasarov and Hoffmann (2020), most of the existing literature on moral licensing has overlooked the role of social influences.

In the present paper, we aimed to extend the literature on this topic by investigating the cross-domain moral licensing and moral consistency effects that may be evoked by reference to recent collective efforts to contain the COVID-19 pandemic. These efforts, and the measures and restrictions they entailed, were extensively debated in Italy (as in other nations, e.g., Druckman et al., 2021), polarizing citizens between those who saw them as necessary and useful in dealing with the emergency (Conway et al., 2020; Beria and Lunkar, 2021), and those who saw them as a collective nuisance or even as a threat to their freedom and identity (Conway et al., 2020; Beria and Lunkar, 2021). We expected that a counterfactual message evoking past efforts to contain the COVID-19 pandemic as a moral credit (i.e., presenting them as an action without which an immoral negative outcome would have occurred) would trigger an inter-domain moral licensing effect in the latter group, as these citizens would be less inclined to support other future policies, particularly when associated with the prospect of further economic costs and hardships. Conversely, we expected that the same counterfactual would trigger an inter-domain moral consistency effect in people who agreed with and endorsed the measures undertaken to contain the pandemic (e.g., mask mandates, restrictions, vaccination policies). Being exposed to a counterfactual of this type would bolster their moral identity and, in turn, lead them to be more willing to engage in further collective economic efforts aimed at the common good, such as climate mitigation.

### Overview

Starting from the above, in our research we presented participants with upward counterfactuals stating that, were it not for past public policies expenses due to the COVID-19 pandemic, further economic efforts to implement climate change policies could have been adopted. We expected exposure to such counterfactuals to reduce support for the adoption of a climate change mitigation policy. This would be the case because a negative, unexpected, and uncontrollable event such as the COVID-19 pandemic provides a very accessible and convenient excuse to participants considering the opportunity to adopt the climate change policy.

In two studies, we investigated to what extent, and under what conditions, counterfactual communication on COVID-19 and related public expenses would affect support for the future adoption of climate change policies. We presented participants with a simulated political debate scenario, where two opposing parties discussed a climate change policy.

In Study 1, we measured the effectiveness of upward counterfactual messages stating that the economic costs of a climate change policy would be affordable, were it not for past public expenses. The scenario was manipulated to include (or not include) the COVID-19 pandemic as the primary motivation for those past expenses. We expected that the counterfactual messages would be more effective, leading to lower support for the climate change policy, when the COVID-19 pandemic was mentioned. Since COVID-19 is a break from “normal” affairs, counterfactual messages focused on it would provide a convincing counterfactual excuse for the unwillingness to support climate action. Such an expectation, which will be formalized in the following section, has theoretical basis in the cross-domain moral licensing effects that have been extensively investigated in the existing literature (e.g., Mazar and Zhong, 2010; Tiefenbeck et al., 2013; see also Theoretical Framework above).

In Study 2, we tested whether the persuasiveness of the upward counterfactual employed in Study 1 would be increased or decreased by the addition of a downward counterfactual hinting at the adoption of the costly anti-COVID measures as a moral credit or, more precisely, as an “immoral road not taken” (i.e., “If we had not imposed restrictions, the number of sick and dead citizens would have been greater”). Put differently, we investigated whether the effect of the upward counterfactual would be boosted or, conversely, hindered, by combining such counterfactual excuse with a downward counterfactual presenting past efforts to deal with the COVID-19 pandemic as a moral credit, that is, as something that prevented an immoral outcome, such as a worse public health situation. We expected that exposure to downward counterfactuals of this type would trigger two opposite outcomes, depending on the participants’ endorsement of the anti-COVID-19 measures the message referred to. Participants with a lower endorsement of the anti-COVID-19 measures would show a form of inter-domain moral licensing (Mazar and Zhong, 2010; Miller and Effron, 2010), “exploiting” the economic sacrifices endured to deal with the pandemic as an excuse to drop their support for further economic sacrifices to implement the climate change policy. On the contrary, participants with a lower endorsement of the anti-COVID-19 measures would show a form of inter-domain moral consistency (Conway and Peetz, 2012), seeing the future engagement in collective economic efforts to address climate change as a continuation of past collective efforts to curb the spread of the pandemic.

## Study 1

In Study 1, participants read a simulated political debate on the adoption of a climate change policy, consisting of two political statements from two different parties, one advocating for the adoption of the policy and the other responding with a counterfactual message arguing that the policy could have been adopted, if only other expensive collective efforts had not been already undertaken in the past. Before reading the scenario, participants were randomly attributed to two different conditions providing different scenarios as the backdrop of the debate. One scenario referred to past public expenses made within the context of the COVID-19 pandemic in a fictional country. The other scenario also referred to past public expenses in a fictional country, but without referring to the COVID-19 pandemic. We formulated the following hypothesis:

*Hypothesis 1*: Participants who read an upward counterfactual message in a scenario in which the COVID-19 pandemic is present support a climate change policy less than participants who read the same message in a scenario in which reference to the COVID-19 pandemic is absent.

This would indicate that the pandemic (and the collective response to it) can be successfully used as the focus of a counterfactual excuse not to engage in further collective endeavors, such as climate change mitigation.

### Methods

#### Participants and procedure

One hundred forty-nine individuals (47.3% males and 52.7% females, age *M* = 40.3, *SD* = 17.1) took part voluntarily in the study. The participants were recruited using a snowball sampling technique. Prospective participants were contacted by students through personal contacts and social media in December 2021. Participation took place online through the Qualtrics platform, and required approximately 15 min. After reading some introductory information, participants were presented with two alternative versions of a scenario about past public expenses in a fictional country, and only in one of them past public expenses were referred to the COVID-19 pandemic. The two versions of the scenario were around 70 words long (for the full text see Table 1, upper pane). Afterwards, participants were presented with a message from the spokesperson of Party A, advocating the reduction of greenhouse emissions through a national plan for renewable energies, and a message from the spokesperson of Party B, arguing against such plan with upward counterfactual sentences. The gist of all counterfactual sentences was that that the climate change plan could have been adopted, if only the country had not already had to face such large public expenditure (for the full text see Table 1, lower pane). After reading the debate extract, all participants were asked to respond to a series of questions.

**Table 1:** Full text of the alternative versions of the scenario and the following debate between Party A and Party B (Study 1).

Past public expenses	
Focus on COVID-19	Absence of focus on COVID-19
Imagine you live in a country where political elections are going to take place soon. The fictional country has been experiencing economic welfare. In the last few years, the economy has grown, even during the COVID-19 pandemic, and a few months before the elections the trend is still positive, despite the costs incurred to tackle the health emergency. The state finances are close to balance and the predictions about the national debt are positive.	Imagine you live in a country where political elections are going to take place soon. The fictional country has been experiencing economic welfare. In the last few years, the economy has grown and a few months before the elections the trend is positive. The state finances are balanced and the predictions about the national debt are positive.
Future public expenses	
Below are two statements describing the respective positions of Party A and Party B on the issue of climate change policies. The messages are excerpts of declarations by the two parties’ spokespeople.	
**Party A’s message**	
Our position is that the fight against global warming must be addressed as soon as possible, making an important effort to reduce greenhouse gas emissions. We are therefore proposing a major national plan to fully convert electricity production from traditional fossil sources to renewable sources. This will be done by phasing out the current coal, oil and gas plants and replacing them with wind farms, hydroelectric plans, and solar panels. If we adopt this plan, we will obtain benefits for the environment and make our country’s energy production completely sustainable.	
**Party B’s message**	
Our position is that we could have adopted the national plan proposed by our opponents, if only we had not already incurred huge costs. If the country had not already spent a lot of money to support the economy, we could have adopted this plan. We could have closed old power plants and converted the energy sector if only we had not already lost many jobs because of the economic crisis.	

#### Measures

##### Support for the climate change policy

The support for the proposed policy was measured using the following three items (Bertolotti et al., 2021): “To what extent do you agree with Party A’s plan on renewable energy?,” rated on a 7-point scale ranging from 1 (“fully disagree”) to 7 (“fully agree”); “Which priority would you attribute to this energy plan in the current political agenda?,” rated on a similar 7-point scale ranging from 1 (“low priority”) to 7 (“high priority”); “How much national funds would you invest in this energy plan in percentage?,” rated on a 7-point scale ranging from 1 (“less than 1%”) to 7 (“More than 20%”). The three item scores (Cronbach’s α = 0.797) were averaged into a single policy support index.

##### Climate change belief

Participants’ belief in climate change was measured with two items, adapted from previous research on climate change beliefs and attitudes (ESS, 2016): “To what extent do you think that the world’s climate is changing?,” rated on a 7-point scale ranging from 1 (“Not at all”) to 7 (“Very much”); “Do you attribute climate change to natural or human factors?,” rated on a 7-point scale ranging from 1 (“Natural factors”) to 7 (“Human factors”), plus an additional option (“The world’s climate is not changing,” coded as 0). The two item scores, *r* (269) = 0.456, *p* < 0.001, were averaged into a single climate change belief index.

##### Biospheric values

As a measure of endorsement of biospheric values (De Groot and Steg, 2008), participants were asked to rate, on a 5-point scale, the importance given to “Protecting the environment” and “Respecting the Earth,” from 1 (“Against my principles”) to 5 (“Very important”). The two items, *r* (149) = 0.753, *p* < 0.001, were then averaged into a single biospheric values index.

##### Socio-demographic variables

These variables included age, gender, education, and profession.

### Results

#### Preliminary analyses

Participants were on average moderately supportive of the climate change policy, *M* = 4.68, *SD* = 1.23. Support for climate change policies (*M* = 4.68, *SD* = 1.23) was positively correlated with belief in climate change (*M* = 5.85, *SD* = 1.29), *r*(147) = 0.423, *p* < 0.001, and the endorsement of biospheric values (*M* = 4.45, *SD* = 0.67), *r*(147) = 0.340, *p* < 0.001.

#### Predictors and potential moderators of support for the climate change policy

To test our hypothesis, we ran a regression model using PROCESS (Hayes, 2022, Model 1), with participants’ support as the dependent variable and the presence/absence of focus on COVID-19 as a predictor (contrast-coded −1 for the absence of COVID-19 emergency and +1 for the presence of it). Additionally, we explored the possibility that participants’ environmental commitment, measured as either belief in climate change or the endorsement of biospheric values, would moderate this effect (e.g., van den Broek et al., 2017). Two separate regression models were run, to account for the different psychometric properties of the two measures, in particular the substantial skewness of the climate change belief measure. Finally, we included participants’ political orientation as a covariate.

**Figure 1:**
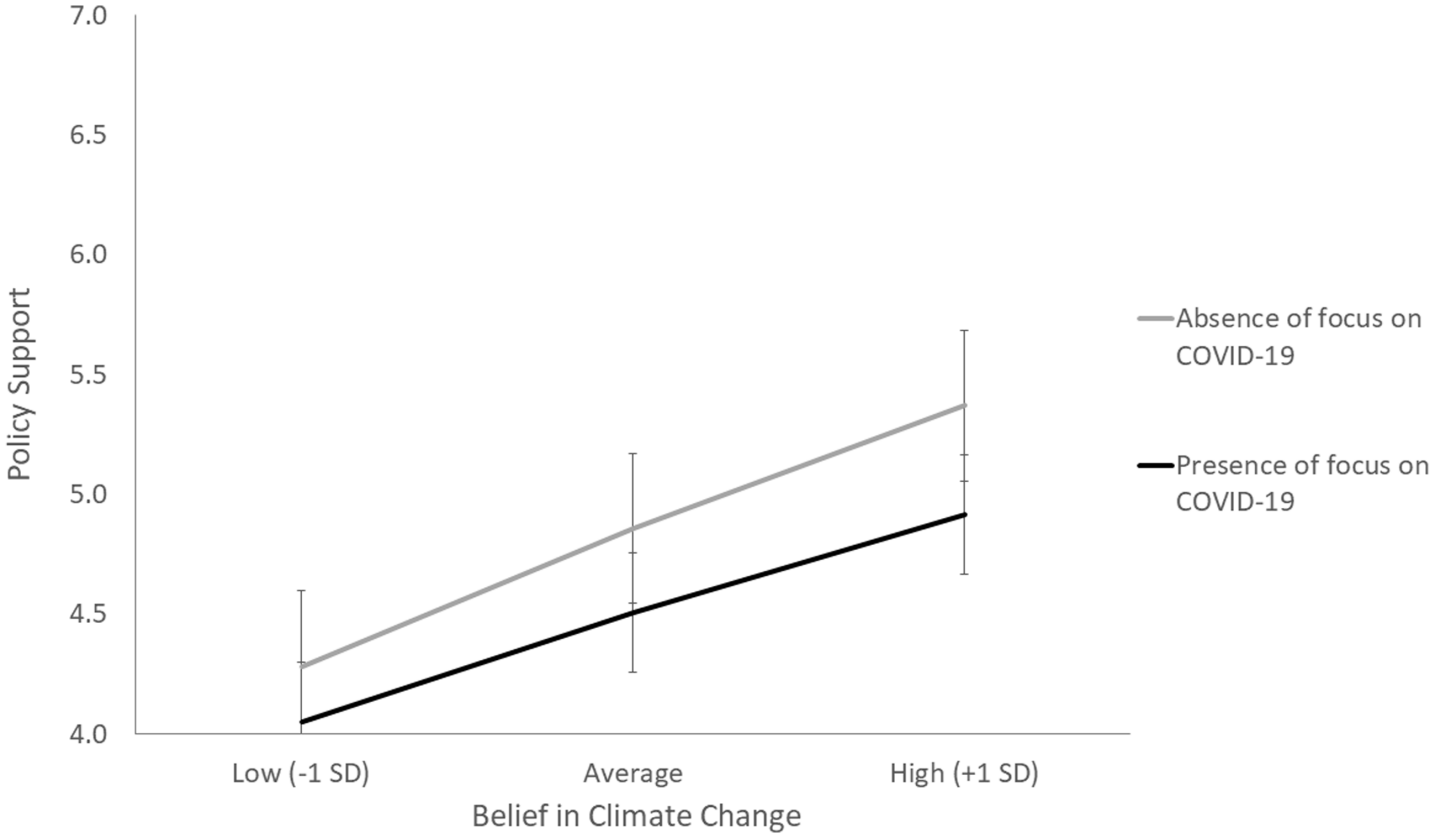
Policy support as a function of the experimental manipulation of the scenario and participants’ belief in climate change.

The scenario manipulation showed a trend toward statistical significance, *B* = −0.17, *SE* = 0.09; *t* = − 1.93, *p* = 0.055, 95% CI [− 0.35; 0.01]. Participants’ support was significantly lower in the COVID-19 condition (*M* = 4.49, *SD* = 1.21) than in the control condition, where reference to COVID-19 was absent (*M* = 4.89, *SD* = 1.23), *t* (147) = 2.00, *p* = 0.048. This result supported our *H1*. Belief in climate change also had a strong significant main effect on the support for the climate change policy, *B* = 0.4, *SE* = 0.07; *t* = 5.58, *p* < 0.001, 95% *CI* [0.26; 0.54], with higher belief in climate change leading to higher support for the climate change policy. The interaction between belief in climate change and the scenario manipulation was however not significant, *B* = − 0.04, *SE* = 0.07; *t* = − 0.63, *p* = 0.532, 95% *CI* [− 0.19; 0.1], showing that the main effect of the scenario manipulation was not moderated by belief in climate change. Finally, the effect of the political orientation covariate was also statistically significant, *B* = −0.05, *SE* = 0.03; *t* = 2.04, *p* = 0.043, 95% *CI* [− 0.1; −0.01], showing greater support for the climate change policy among left-wing participants. Figure 1 shows the main effect of the scenario manipulation at different levels of belief in climate change.

We then replicated the same regression model, but this time with biospheric values as the moderator of the effect of the scenario manipulation. We found a main effect of the scenario manipulation approaching statistical significance, *B* = −0.16, *SE* = 0.09; *t* = −1.72, *p* = 0.087, 95% *CI* [−0.35; 0.02]. We also found a significant main effect of the endorsement of biospheric values, *B* = 0.64, *SE* = 0.14; *t* = 4.42, *p* < 0.001, 95% *CI* [0.35; 0.92], while, again, the interaction with the scenario manipulation was not significant, *B* = −0.18, *SE* = 0.14; *t* = −1.22, *p* = 0.223, 95% *CI* [−0.46; 0.11], showing that the effect of the scenario manipulation was not moderated by the endorsement of biospheric values. The full results of the two regression models are reported in Table 2.

**Table 2:** Hierarchical regression models of support for the climate change policy (Study 1).

	Model 1						Model 2					
	*B*	S.E.	*t*	*p*	LL 95% *CI*	UL 95% *CI*	*B*	S.E.	*t*	*p*	LL 95% *CI*	UL 95% *CI*
(Constant)	5.062	0.207	24.441	0.000	4.65	5.47	5.079	0.212	23.983	0.000	4.66	5.50
Political Orientation	−0.053	0.026	2.039	0.043*	−0.011	0.000	−0.057	0.028	2.135	0.035	−0.11	0.00
Counterfactual Focus	−0.175	0.090	1.935	0.055	−0.35	0.00	−0.161	0.094	1.972	0.049*	−0.35	0.00
Belief in Clim. Ch.	0.400	0.090	5.578	0.000**	0.26	0.54						
Biospheric values							0.638	0.144	4.42	0.000**	0.35	0.92
*CF* Focus × Belief in Clim. Ch.	−0.046	0.073	0.626	0.532	−0.19	−0.10						
*CF* Focus × Biospheric values							−0.177	0.145	1.22	0.223	−0.46	0.11

*p < 0.05; **p < 0.001.

To sum up, our results corroborated our *H1* hypothesis. The counterfactual message employed by the spokesperson of party B resulted in lower support for the climate change policy when participants had previously read that past public expenses had been devoted to fight the COVID-19 emergency than when they had simply read a message on past public expenses. Therefore, the counterfactual argument against the adoption of the climate change policy employed by Party B was more effective when the “abnormal” element of the pandemic had been previously introduced in the scenario than when it had not. This effect was not moderated by participants’ beliefs and values related to climate change.

## Study 2

In Study 2, we tested whether the persuasiveness of the counterfactual message against the adoption of a climate change policy would be increased, or instead reduced, in a new condition in which party A and Party B discussed the pandemic-related efforts before discussing the climate change policy, with Party B claiming that “if costly anti-COVID measures had not been enacted, we would have suffered a much higher human cost.” The anti-COVID measures described in the scenario were reminiscent of those adopted in Italy (i.e., participants’ own country of residence), and included social distancing, use of personal protective equipment, travel restrictions, etc.…). We expected that this introduction of a downward counterfactual message hinting at an “immoral road not taken” (Effron, 2014) would trigger either a moral licensing or a moral consistency effect on support for the climate change policy, depending on participants’ own stance on the anti-COVID measures.

Consequently, we formulated the following hypothesis.

*Hypothesis 2*: After exposure (versus non-exposure) to downward counterfactuals on past economic efforts to tackle a looming problem with consequences on a wide range of levels (such as the COVID-19 pandemic), participants with low endorsement of measures aimed at addressing such a problem show lower support for future economic efforts to implement a climate change policy (*H2*a, moral licensing effect). Conversely, after exposure (versus non-exposure) to the same downward counterfactuals, participants with high endorsement of the abovementioned measures show higher support for economic efforts to implement a climate change policy (*H2*b, moral consistency effect).

### Methods

#### Participants and procedure

One hundred forty-seven Italian participants (47.2% males, 52.8% females, and 0.7% other, age *M* = 40.4, *SD* = 16.2) took part voluntarily in the study. The procedure was the same of Study 1, with participants being presented a fictional debate between two parties.

Participants in the experimental condition (*N* = 68) read two excerpts of the debate between Party A and Party B. In the first excerpt, the two Parties discussed the public health measures adopted in the country to fight the COVID-19 pandemic. Party A stressed the importance of the economic expenses made to increase social distancing, the use of personal devices and the restrictions on mass gatherings, including shutting down pubs and restaurants, and public events. Party B further remarked that “if those expenses had not been made, there would have been much more severe negative consequences on citizens’ health.” The full text of this exchange is reported in Table 3. Then, participants were presented the same exchange between Party A and Party B employed in Study 1, with Party B again arguing that the renewable energy plan would have been possible, if only the nation had not already sustained severe economic repercussions from the adoption of anti-COVID measures. Participants in the control condition (*N* = 79) read only this second excerpt. All participants were then asked to answer some questions on what they had just read. A small number of participants (*N* = 16) failed an attention check and were consequently excluded from the main analyses.

**Table 3:** Additional text presented to participants in the experimental condition (Study 2). Counterfactuals are highlighted in italics.

Below are two statements describing the respective positions of Party A and Party B on the issue of public health policies. The messages are excerpts of declarations by the two parties’ spokespeople.
**Party A**
Regarding the issue of health, our party intends to continue along the line followed in recent months by most of the world’s governments to tackle the Covid-19 pandemic. We intend to finance the development of new medicines, to treat existing cases, and to continue testing vaccines to prevent the spread of the virus. As long as these treatments are available, however, we will have to continue to maintain existing measures, such as social distancing, the use of personal protective devices and restrictions on gatherings in public places. This could include other periods of closure for certain types of venues, the suspension of public events such as matches, shows and concerts, and other restrictions on transport and freedom of movement.
**Party B**
Our stance on health is similar to that of our opponents. *If we had not incurred serious economic costs in these months to tackle the pandemic, the consequences on citizens’ health would have been worse*. If we had not imposed restrictions on many businesses, with the loss of jobs and the closure of many companies, the number of sick and dead citizens would have been greater. If we had not increased the public debt by worsening the state accounts, the health situation would be worse.

#### Measures

The same items employed in Study 1 were used to measure support for the climate change policy, Chronbach’s *α* = 0.745, biospheric values, *r* (128) = 0.698, *p* < 0.001, and political orientation. Basic socio-demographic information was collected, as well.

##### Endorsement to anti-COVID measures

The respondents’ opinion about anti-COVID19 policies was assessed using two items: “I think that it is fair that the Government limits its citizens’ freedom of movement for health reasons”; and “Related to COVID-19 pandemic, the restrictions imposed by the Government to contain the spread of the virus are appropriate.” Agreement with each statement was recorded on a 7-point scale ranging from 1 (“Completely disagree”) to 7 (“Completely agree”). The two item scores, *r* (126) = 0.580, *p* < 0.001, were averaged into a single anti-COVID measures endorsement index.

### Results

#### Preliminary analyses

As in Study 1, participants were on average moderately supportive of the climate change policy, *M* = 5.00, *SD* = 1.13. Support was positively correlated with both biospheric values (*M* = 4.59, *SD* = 0.56), *r* (128) = 0.408, *p* < 0.001, and endorsement of anti-COVID measures (*M* = 5.02, *SD* = 1.28), *r* (127) = 0.296, *p* = 0.001, and negatively correlated with political orientation, *r* (128) = −0.218, *p* < 0.001, indicating that left-leaning participants supported the proposed policy more than right-leaning participants.

#### Predictors of support for the climate change policy

To test our research hypothesis, we ran a regression model with the experimental condition as the main predictor, the endorsement of anti-COVID policies as a moderator, and participants’ support for the climate change policy as the dependent variable. Biospheric values and political orientation were included in the model as covariates, based on the findings form Study 1 where both variables independently predicted support, but did not interact with the manipulated message.

The results of the full regression model are reported in Table 4. The strong positive association between biospheric values and support for the climate change policy found in Study 1 was found also in Study 2, *B* = 0.66, *SE* = 0.17; *t* = 3.90, *p* < *0*.001, 95% *CI* [0.32; 1.00], whereas no significant association with the endorsement of anti-COVID measures, *B* = −0.36, *SE* = 0.21; *t* = 1.69, *p* = *0*.094, 95% *CI* [−0.79;0.06], or political orientation, *B* = −0.02, *SE* = 0.03; *t* = 0.61, *p* = *0*.543, 95% *CI* [−0.08; 0.04], emerged.

**Table 4:** Hierarchical regression model of support for the climate change policy (Study 2).

	*B*	S.E.	*t*	*p*	LL 95% *CI*	UL 95% *CI*
(Constant)	2.108	0.861	2.450	0.026	0.405	3.812
Biospheric values	0.660	0.170	3.895	0.001**	0.325	0.996
Political orientation	−0.019	0.031	0.610	0.543	−0.080	0.042
Presence versus absence downward *CF*	−0.033	0.179	0.184	0.854	−0.388	0.322
Endorsement of anti-COVID measures.	−0.361	0.214	1.688	0.094	−0.785	0.063
Presence versus absence downward *CF* × Endorsement of anti-COVID measures	0.365	0.142	2,575	0.011*	0.084	0.645

*p < 0.05; **p < 0.001.

**Figure 2:**
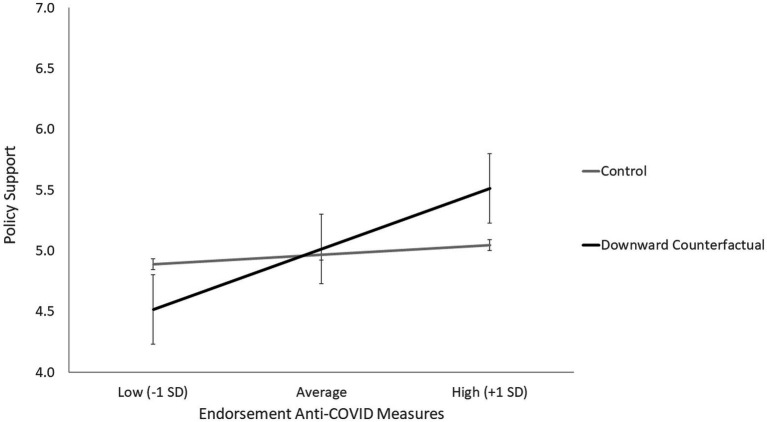
Policy support as a function of the experimental manipulation and participants’ endorsement of anti-COVID measures (Study 2).

Results showed no main effect of the experimental condition, *B* = −0.03, *SE* = 0.18; *t* = 0.18, *p =* 0.854, 95% *CI* [−0.39; 0.32], while the predicted interaction effect was found, *B* = 0.36, *SE* = 0.14; *t* = 2.58, *p* = 0.011, 95% *CI* [0.08; 0.64]. Consistent with our *H2*a, participants with lower endorsement of the anti-COVID measures showed lower support for the policy in the experimental condition than in the control condition (Figure 2), as indicated by the negative conditional effect of the manipulation at low levels (−1 *SD*) of endorsement, *B* = −0.50, *SE* = 0.26; *t* = 1.95, *p* = *0*.054, 95% *CI* [−1.00; 0.01]. Conversely, and consistent with our *H2*b, participants with higher endorsement of the anti-COVID measures showed higher support for the policy in the experimental condition than in the control condition, although the conditional effect at high levels of endorsement only approached significance, *B* = 0.43, *SE* = 0.25; *t* = 1.71, *p* = *0*.091, 95% *CI* [−0.07; 0.93].

To sum up, the results of Study 2 confirmed our research hypothesis. The introduction of a downward counterfactual scenario according to which things could have been worse if it were not for their past effort to curb the COVID-19 pandemic determined opposite reactions as regards the adoption of a climate change policy, depending on participants’ endorsement of anti-COVID measures. Among participants who did not consider the anti-COVID measures very useful and effective, the moral credit evoked by the “immoral road not taken” scenario hindered support for the climate change policy, thus showing evidence of a moral licensing effect. When reminded that things could have been worse if it were not for their past effort to curb the pandemic, these participants tended to refrain from committing to other future collective efforts to deal with the issue of climate change. Conversely, among participants who considered the anti-COVID measures useful, exposure to the same scenario increased support for the policy, suggesting the presence of a moral consistency effect. When reminded of the moral value of their past deeds (i.e., the costly, but life-saving measures to curb the pandemic), these participants extended the same commitment to other future efforts to save the planet.

## General discussion

Our results showed that, overall, upward counterfactual economic arguments focused on the unforeseen public expenses to curb the COVID-19 pandemic (i.e., “We could have done it, if it were not for COVID-19”) can be used as an excuse to convince citizens to withdraw their support for future climate mitigation efforts. Upward counterfactual arguments were indeed more convincing when presented in the context of an extra-ordinary, routine-breaking event such as the COVID-19 pandemic, compared to when they merely referred to past economic expenses (Study 1). When this type of excuse was combined with a downward counterfactual argument focused on the negative public health consequences avoided by such past expenses, however, a moderation effect of participants’ attitude toward anti-COVID-19 measures emerged (Study 2). Among participants with low endorsement of these measures they reduced support for a proposed climate change policy, which is consistent with an inter-domain moral licensing effect. Conversely, among participants with high endorsement of the measures they increased support for the climate change policy, which is consistent with an inter-domain moral consistency effect.

These findings advance our understanding of communication in the domain of climate change policies, and in particular the effects of economic arguments against said policies, and how downward counterfactuals can trigger moral licensing or consistency effects across different domains.

First, whereas past research already indicated that economic arguments based on the future costs of climate change policies can be critical in undermining agreement with them (Bertolotti et al., 2021), results on how communication about public expenses sustained in the past can affect decisions about future public expenses were still missing. In our studies, counterfactuals regarding the economic and human costs of governmental measures imposed to handle the COVID-19 pandemic were used to alter support for a future policy dealing with climate change. Past research has already established that counterfactual thinking can significantly affect reasoning and evaluative processes (Epstude and Roese, 2008), as individuals (and groups, Milesi & Catellani, 2011) are able to critically reassess past events, particularly those with negative, unexpected and unwanted outcomes, and imagine how things could have gone better. Further research has shown that this process can be strategically triggered by communication, focusing the audience’s attention on what a certain actor could or should have done in the past, to change the audience’ attributions and attitudes (Bertolotti et al., 2013; Bertolotti and Catellani, 2018; Catellani et al., 2021). Counterfactual excuses (Markman and Tetlock, 2000; Catellani and Bertolotti, 2014) can successfully deflect responsibility from oneself to a convenient external target, which can be consequently blamed for undesirable results, or indefensible behavior. Findings from our Study 1 indicate that the same mechanism can be used not only to excuse past behavior, but also to excuse future behavior (i.e., inaction in the daunting challenge to tackle global climate change). Furthermore, we found that the COVID-19 pandemic, being a negative, unforeseen, and uncontrollable event, provides a very convenient focus (Gerstenberg et al., 2012) for this type of counterfactual communication. Remarkably, this effect seemed to hold for all participants, including those with high endorsement of biospheric values, indicating that this type of argument might work not only for those who have relatively little interest in the issue of climate change (including the so-called climate skeptics, Maibach et al., 2011), but also for those who would otherwise strongly support all forms of climate action.

Second, our results on the effects of downward counterfactual arguments complement the existing research on moral licensing effects and their moderating individual factors. They show that counterfactual communication can be used to elicit a cross-domain moral licensing effect, but also that this effect can be substantially altered, and even reversed, by the pre-existing convictions on the domain where a moral credit is claimed. The factor we considered here, that is, the endorsement of the set of preventive measures adopted to counter the spread of the COVID-19 pandemic, might be seen as corresponding, at the collective action and policy level, to the intrinsic motivation for individual behavior investigated in previous research on the moderators of the moral licensing effect (Jordan et al., 2011; Conway and Peetz, 2012; Simbrunner and Schlegelmilch, 2017). Reference to a “immoral road not taken” can effectively convey one’s reluctance and skepticism on a previous collective effort to a future one, resulting in disengagement and reduced support for it. If, instead, the past commitment to one issue is high, the moral credit framing conveyed by the counterfactual argument seems to make the moral norms underlying the former effort more salient, and their relevance to another domain more compelling.

As counterfactual thinking is known to enhance motivation and preparation in future self-relevant behaviors (Epstude and Roese, 2008; Ferrante et al., 2013; Hammell and Chan, 2016; Roese and Epstude, 2017), this mechanism might help explaining the inconsistent findings of past research on the consequences of claiming moral credits for past actions on future intentions and behaviors, particularly in the pro-environmental domain (Gholamzadehmir et al., 2019; Urban et al., 2019). Counterfactual communication might focus the attention on the moral dimension that the public health and environmental crisis have in common and empower citizens by making one averted disaster salient, helping them realize that another one can be prevented (thus representing an instance of the so-called “*spillover effect*,” Thøgersen and Crompton, 2009).

Our research has some limitations. A first limitation is the hypothetical nature of the scenario employed, which reduced the ecological validity of our findings. Furthermore, past research found that moral licensing effects appear to be weaker in hypothetical decisions than in real-world decisions (Batson and Thompson, 2001; Blanken et al., 2015). This implies that we might have been able to observe even stronger effects, if we had employed a more realistic scenario, and measured participants’ actual decisions in it. Future research might explore this possibility, using additional measures to assess the intra-personal processes associated with these decisions (e.g., attitudinal changes, self-justification, or moral image bolstering, Effron, 2014; Barkal et al., 2015). A second limitation derives from the non-representative samples used in our two studies. Our research was conducted in Italy, which, as a YouGov survey showed (YouGov, 2020), is one of the countries where the dualism between prioritizing environmental transition vs. economic recovery was perceived as most critical, which might have made the Italian participants of our studies very sensitive to the type of communication employed in our experimental scenario. Future research might replicate a similar paradigm in different national and cultural environments, where different types of arguments in favor and against climate change policies are likely accessible to citizens (e.g., based on ideological or value-related polarization, Wolsko et al., 2016; Leiserowitz et al., 2021).

## Conclusion

To conclude, our results show that counterfactuals regarding the expenses for facing the COVID-19 pandemic can affect citizens’ attitudes toward an equally urgent but seemingly unrelated item in the global agenda, that is how to handle the environmental crisis. As the COVID-19 pandemic and its social and economic consequences are likely to continue dominating the public sphere for some time, we should expect it to interfere with climate change communication also in the future. The results of our research might give policy makers, governments, and public and private actors some insights on how to confront this additional hurdle to the adoption of urgently needed climate change policies. As our results demonstrated, whereas communication increasing the salience of recent collective economic sacrifices might hinder support for climate change policies, getting citizens to know that their past efforts were morally rightful, and did make a difference, might help them find new motivations to act responsibly also in the environmental domain.

## Data availability statement

The raw data supporting the conclusions of this article will be made available by the authors, without undue reservation.

## Ethics statement

The studies involving human participants were reviewed and approved by CERPS (Ethics Committee for Research in Psychology) Catholic University of the Sacred Heart, Milan. The patients/participants provided their written informed consent to participate in this study.

## Author contributions

MB: conceptualization, study design, data analysis, writing and editing. LV: data analysis, writing and editing. PC: supervision, conceptualization, study design, writing and editing. All authors contributed to the article and approved the submitted version.

## Conflict of interest

The authors declare that the research was conducted in the absence of any commercial or financial relationships that could be construed as a potential conflict of interest.

## Publisher’s note

All claims expressed in this article are solely those of the authors and do not necessarily represent those of their affiliated organizations, or those of the publisher, the editors and the reviewers. Any product that may be evaluated in this article, or claim that may be made by its manufacturer, is not guaranteed or endorsed by the publisher.
